# Efficacy of Pilocarpine and Bromhexine in Improving Radiotherapy-induced Xerostomia

**DOI:** 10.5681/joddd.2013.015

**Published:** 2013-05-30

**Authors:** Farid Abbasi, Sareh Farhadi, Mostafa Esmaili

**Affiliations:** ^1^Associate Professor, Department of Oral Medicine, Faculty of Dentistry, Shahed University, Tehran, Iran; ^2^Assistant Professor, Department of Oral and Maxillofacial Pathology, Faculty of Dentistry, Shahed University, Tehran, Iran; ^3^Assistant Professor, Department of Oral Medicine, Faculty of Dentistry, Shahed University, Tehran, Iran

**Keywords:** Bromhexine, pilocarpine, radiotherapy, xerostomia

## Abstract

***Background and aims.*** Xerostomia is one of the most common complications of head and neck radiotherapy. The aim of this study was to evaluate and compare the efficacy of pilocarpine and bromhexine in improving radiotherapy-induced xerostomia and its associated symptoms.

***Materials and methods.*** In this single-blind, randomized crossover study, pilocarpine and bromhexine tablets were used by twenty-five patients suffered from xerostomia, with a medical history of head and neck radiotherapy. At step A, the patients were treated with pilocarpine for 2 weeks. In addition, they were asked to take bromhexine for 2 weeks with a one-week washout period. At step B, the inverse process was conducted (first bromhexine, then pilocarpine). Whole resting saliva was collected from patients before and after receiving each medication by precise measurements. Then, efficacy of the two drugs in the treatment of xerostomia and its related oral complications was evaluated using questionnaires by Dichotomous format. The results were statistically analyzed using t-student and Fisher’s exact and chi-squared tests. Statistical significance was set at P<0.05.

***Results.*** The difference between saliva secretion rates before and after medications was not significant for bromhexine users at two steps of the study (P=0.35); however, it was significant for pilocarpine users (P=0.0001). Users of both drugs showed significant differences in improvement of xerostomia, chewing, swallowing, tasting and mouth burning.

***Conclusion.*** Pilocarpine is probably more effective in improving xerostomia and its associated problems compared with bromhexine, although the use of the latter was also shown to ease some of the consequences of radiotherapy in the head and neck region.

## Introduction

The importance of saliva in protecting the oral cavity becomes more apparent when malfunction of salivary glands results in xerostomia.^1^ The problems experienced by patients may include a persistent dry or burning sensation, eating difficulties, diminution in taste acuity, discomfort during speaking, mucosal infections, denture intolerance and bacterial sialadenitis.^2^ These symptoms reflect not only the mechanical function (moisture, irrigation and lubrication) of saliva, but also its buffering properties.^3^

Nowadays, consumption of antidepressant drugs, radiotherapy of the head and neck region and some systemic diseases, such as diabetes mellitus, are some of the conditions that induce xerostomia.^4,5^ Radiotherapy is used for suppression of malignant cells but injury to normal cells can be inevitable. Most of the patients with a history of head and neck radiotherapy complain of some degrees of xerostomia due to presence of salivary glands in the radiation field. Hence, destruction of gnathic bone and oral mucosa might be notable.^6-8^ A reduction in salivary flow rate and decrease of its pH is paralleled with a change in saliva competence and shifting of oral microflora to cariogenic bacterial spices.^9^ Therefore, discomfort in chewing, swallowing, speech, sleep and also progressing of periodontal diseases and dental caries probably occur in the presence of xerostomia.^10-12^

Studies have led to four therapeutic suggestions for xerostomia: preventive and symptomatic treatments, local and systemic stimulation.^13-15^ In relation to systemic medications, bromhexine is recognized as a diluting agent of mucous secretions in respiratory tract and pilocarpine is a parasympathomimetic medication acting as salivary and lacrimal secretion stimulator. Many studies have verified that pilocarpine can provide clinically significant symptomatic relief to patients suffering from radiotherapy-induced xerostomia^10-12^ and also in cases of Sjögren syndrome;^16^ but there are few studies about efficacy of bromhexine in these cases.^13-15^ Furthermore, we could not find any reports making comparisons between efficacy of pilocarpine and bromhexine in these conditions.

Therefore, this study was designed to evaluate and compare the efficacy of pilocarpine and bromhexine in improving radiotherapy-induced xerostomia and its associated symptoms.

## Materials and Methods

This single-blind, randomized crossover study evaluated twenty-five patients of Imam Reza Hospital of Kermanshah, Iran, who suffered from xerostomia and their medical history showed head and neck radiotherapy, corresponding to similar studies in this manner.^14,17^ All the patients were over 18 years of age and had been treated with more than 4500cGy of radiation dose in 6.5 weeks more than 6 months previously. Patients with recurrent cancer, diabetes mellitus, asthma, consumption of antidepressant drugs and sensitivity to pilocarpine and bromhexine were excluded from the study. After taking an informed consent, the study was planned in 2 steps of A and B in order to reduce experimental errors. At step A, the patients were advised to use 5-mg pilocarpine tablets (Mahya Daroo Co.) 4 times daily for 2 weeks. After 2 weeks, the patients were asked to stop taking the drug for one week in order to obliterate the pharmacologic effects of the drug (wash-out period).^18^ Then, they were asked to take 8-mg bromhexine tablets (Mucolin tablets, Tolidaroo Co.) 4 times daily for 2 weeks. The inverse process was conducted at step B (first bromhexine, then pilocarpine). The patients’ whole resting saliva was collected and measured precisely before and after every course of medication by two experts: one oral medicine specialist and one student of dentistry who was trained in this procedure. The resting saliva secretion was measured using spitting methods^19^ and levels of lower than 0.01 mL reflected dysfunction of salivary glands. The patients were not informed about the prescribed drugs as dictated by the single-blind research design.

Then, the patients answered the self-administered questionnaire, during the first visit (zero day) and fourteen days after taking the medication; this was repeated for another drug in the same manner. The questionnaire was designed by a specialist of oral medicine in relation to dichotomous scale, including 15 questions about xerostomia and its oral complications such as swallowing, speech, tasting problems and burning sensation.

Improvement of xerostomia and other oral complications was statistically analyzed by chi-squared and Fisher’s exact tests. Increase in saliva secretion, before and after medication, was analyzed by Student’s t-test. Statistical significance was defined at P<0.05.

## Results

Tables 1 and 2 show the rate of saliva secretion at step A (first pilocarpine, then bromhexine) and B (first bromhexine, then pilocarpine), respectively, in four separate evaluations: before and after first evaluation and before and after second evaluation. Comparisons between the rate of secretion showed no significant differences in bromhexine users (P=0.35) but there were significant differences in pilocarpine users (P=0.0001).

**Table 1:** Mean and SD of saliva secretion at step A (first pilocarpine, then bromhexine) before and after first and second evaluations

Time of evaluation	rate of secretion (mL)
Before first evaluation	0.08±0.02
After first evaluation	0.69±0.27
Before second evaluation	0.08±0.02
After second evaluation	0.11±0.06

**Table 2:** Mean and SD of saliva secretion at step B (first bromhexine, then pilocarpine) before and after first and second evaluations

Time of evaluation	rate of secretion (mL)
Before first evaluation	0.08±0.02
After first evaluation	0.09±0.01
Before second evaluation	0.08±0.02
After second evaluation	0.61±0.23

Furthermore, 28% and 100% of bromhexine and pilocarpine users showed improvement of xerostomia after fourteen days, respectively. Statistical analysis showed significant differences in improvement of xerostomia for users of both medications (P=0.0001).

All the (100%) pilocarpine users and 14.3% of bromhexine users demonstrated improvement of chewing difficulties; similarly, 87.5% of pilocarpine users and 25% of bromhexine users showed improvements in swallowing problems; 100% of pilocarpine users and 14.3% of bromhexine users, reported relief of speech problems and 90.9% of pilocarpine users and 20.8% bromhexine users showed improvements in tasting difficulties. Finally, 100% of pilocarpine users and 66.7% of bromhexine users demonstrated improvements in burning sensation. All the differences mentioned were statistically significant with P-values of 0.0001, 0.04, 0.005, 0.0001 and 0.004 for improvement in chewing, swallowing, speech, tasting problems and burning sensation, respectively.

## Discussion

Radiotherapy of head and neck may result in a decrease in salivary pH and its rate of secretion. Therefore, any discomfort of chewing, swallowing, speech and sleep may occur in the presence of xerostomia. In order to relieve these oral discomforts, salivary stimulating drugs, such as pilocarpine and bromhexine, have been used for some years and their efficacy has been verified in some experimental studies.^10-12^

In the present study, improvement of xerostomia was shown using both medications. Chitapanarux et al^17^ and Ram et al,^20^ in line with this study, reported that pilocarpine has obvious palliative effects on xerostomia and sleep of patients. Previous studies^21-23^ have shown that pilocarpine has a great ability to prevent radiotherapy-induced xerostomia. Haddad et al^24^ showed the preventive effect of both drugs and Wu et al^25^ reported that pilocarpine can improve xerostomia induced by Sjögren syndrome. Although, some researchers have reported that pilocarpine increases saliva secretion,^26,27^ Warde et al, contrary to the results of the present stydy, reported no significant differences in recovery from xerostomia and quality of life between pilocarpine and placebo users.^28^ It seems that use of VAS scale in their study and different frequencies of drug administration can explain this lack of difference.

Notable effects of pilocarpine have been confirmed in improving radiotherapy-induced xerostomia,^10-12,24^ Sjögren syndrome^29^ and immune dysfunction conditions.^30^ Given the efficacy of this medication in increasing saliva secretion, the practitioners have preferred to advise it rather than artificial saliva in these situations;^31^ however, indication of pilocarpine prescription was limited in cases with complete suppression of salivary gland function. On the other hand, there are few scientific reports on bromhexine with definite results. However, Avisar et al^32^ and Frost-larsen et al^33^ showed improvement of xerostomia with administration of bromhexine in cases of Sjögren syndrome.

The present study showed significant differences in all the signs and symptoms of radiotherapy-induced xerostomia using both medications, though pilocarpine showed a more effective role compared with bromhexine. Previous studies^34-36^ have shown significant increases in saliva secretion following pilocarpine administration. Frost-larsen et al,^33^ in line with the present study, reported significant increases in saliva secretion after bromhexine administration but Misawa et al^37^ could not find any significant differences. Different etiologies of xerostomia in these studies can probably explain the situation.

The present single-blind study generally showed the superiority of Pilocarpine to Bromhexine in improving radiotherapy-induced xerostomia but further investigations are necessary to compare the long-term efficacy of these two drugs. In addition, application of quality of life questionnaire in 100-scale VAS might be a more precise evaluation of the situation.

## Conclusion

Pilocarpine is probably more effective in improving xerostomia and its associated problems compared with bromhexine, although the use of the latter was also shown to remove some consequences of radiotherapy in the head and neck region.
